# CX_3_CR1 Mediates the Development of Monocyte-Derived Dendritic Cells during Hepatic Inflammation

**DOI:** 10.3390/cells8091099

**Published:** 2019-09-18

**Authors:** Salvatore Sutti, Stefania Bruzzì, Felix Heymann, Anke Liepelt, Oliver Krenkel, Alberto Toscani, Naresh Naik Ramavath, Diego Cotella, Emanuele Albano, Frank Tacke

**Affiliations:** 1 Department of Health Sciences and Interdisciplinary Research Centre for Autoimmune Diseases, University “Amedeo Avogadro” of East Piedmont, Via Solaroli 17, 28100 Novara, Italy; bruzzi.stefania@hsr.it (S.B.); alberto_toscani@outlook.it (A.T.); naresh.ramavath@uniupo.it (N.N.R.); diego.cotella@med.uniupo.it (D.C.); emanuele.albano@med.uniupo.it (E.A.); 2 Department of Hepatology and Gastroenterology, Charité University Medical Center Berlin, 10117 Berlin, Germany; felix.heymann@googlemail.com (F.H.); frank.tacke@charite.de (F.T.); 3 Department of Medicine III, RWTH-University Hospital Aachen, 52074 Aachen, Germany; apfeiffer@ukaachen.de (A.L.); okrenkel@ukaachen.de (O.K.)

**Keywords:** dendritic cells, liver injury, fractalkine, carbon tetrachloride

## Abstract

Recent evidence suggests that hepatic dendritic cells (HDCs) contribute to the evolution of chronic liver diseases. However, the HDC subsets involved and the mechanisms driving these responses are still poorly understood. In this study, we have investigated the role of the fractalkine receptor CX_3_CR1 in modulating monocyte-derived dendritic cell (moDC) differentiation during liver inflammation. The phenotype of HDC and functional relevance of CX_3_CR1 was assessed in mice following necro-inflammatory liver injury induced by the hepatotoxic agent carbon tetrachloride (CCl_4_) and in steatohepatitis caused by a methionine/choline-deficient (MCD) diet. In both the experimental models, hepatic inflammation was associated with a massive expansion of CD11c^+^/MHCII^high^/CD11b^+^ myeloid HDCs. These cells also expressed the monocyte markers Ly6C, chemokine (C-C Motif) receptor 2 (CCR2), F4/80 and CD88, along with CX_3_CR1, allowing their tentative identification as moDCs. Mice defective in CX_3_CR1 showed a reduction in liver-moDC recruitment following CCl_4_ poisoning in parallel with a defective maturation of monocytes into moDCs. The lack of CX_3_CR1 also affected moDC differentiation from bone marrow myeloid cells induced by granulocyte-macrophage colony stimulating factor (GM-CSF) and interleukin-4 (IL-4) in vitro. In wild-type mice, treatment with the CX_3_CR1 antagonist CX3-AT (150 µg, i.p.) 24 h after CCl_4_ administration reduced liver moDC_S_ and significantly ameliorated hepatic injury and inflammation. Altogether, these results highlight the possible involvement of moDCs in promoting hepatic inflammation following liver injury and indicated a novel role of CX_3_CL1/CX_3_CR1 dyad in driving the differentiation of hepatic moDCs.

## 1. Introduction

In recent years, growing attention has been paid to the importance of myeloid cells in modulating the evolution of both acute and chronic liver injury [1,2]. Although these studies have mainly emphasized the importance of Kupffer cells and monocyte-derived macrophages [3], recent evidence also points to the possible involvement of dendritic cells [4]. 

During homeostasis, hepatic dendritic cells (HDCs) represent a small sub-fraction (less than 1%) of total liver myeloid cells. HDCs are mainly localized in the portal areas, although a recent study in mice has identified them also under the liver capsule [5]. Based on specific membrane markers and functional features, HDCs can be distinguished into plasmacytoid and myeloid or classical subsets. Myeloid HDCs are further sub-grouped in type-1 (CD103^+^/CD11b^−^ in mice; CD141^+^/CD14^−^ in humans) and type-2 (CD103^−^/CD11b^+^ in mice; CD1c^+^/CD14^−^ in humans) cells [4,6]. In healthy livers, HDCs display a predominant immature phenotype characterized by a low capacity to endocytose antigens and stimulate T-lymphocytes. These features, along with the production of interleukin-10 (IL-10), interleukin-27 (IL-27) and kynurenine, contribute to the tolerogenic activity of HDCs in healthy livers [7,8]. However, HDCs expand and activate following hepatic injury, becoming efficient antigen-presenting cells and a source of pro-inflammatory cytokines [9,10,11]. 

The actual role of HDCs in the pathogenesis of liver diseases is still a matter of debate, due to contradictory data obtained in experiments in which HDCs have been either artificially expanded or depleted [12,13,14,15,16]. These conflicting results can be explained by the low specificity of the methods used to modulate HDCs, as well as by the fact that several factors (including intracellular lipid content and the interaction with other cells within the liver modify HDC functions [17,18]. Current view indicates HDC expansion following liver injury) mainly involve the myeloid group [10,13] and that myeloid HDCs are also actively engaged in liver immune response to infections [19]. Nonetheless, a specific identification of the myeloid HDCs involved is uncertain, since CD141^+^ type-1 myeloid HDCs are lowered in patients with advanced chronic liver diseases [20]. Furthermore, CD103^+^ type-1 myeloid HDCs display a hepatoprotective action in mice, and their depletion in Batf3-deficient mice favors the onset of steatohepatitis [15]. In the same vein, we have reported that type-2 myeloid HDCs expressing the fractalkine receptor CX_3_CR1 and producing IL-10 are present in the liver during homeostasis and counteract acute hepatic inflammation [16], while the subsequent chronic liver injury HDCs featuring CX_3_CR1 and an increased TNF-α production contribute to the evolution of steatohepatitis [11]. 

A further element of complexity is given by the fact that—in response to inflammatory stimuli—monocytes can differentiate to a distinct dendritic cell subset called monocyte-derived dendritic cells (moDCs) or inflammatory dendritic cells. MoDCs have several surface markers and functional properties in common with type-2 myeloid dendritic cells, although they develop independently from the transcription factors driving myeloid dendritic cell differentiation [21,22,23]. Furthermore, besides acting as antigen-presenting cells to lymphocytes, moDCs actively produce pro-inflammatory mediators [21,22,23]. In view of the growing importance of moDCs in initiating and modulating innate and adaptive immunity during infections, autoimmune diseases and allograft rejection [21,22], this study investigates the possible implications of moDCs in hepatic inflammation, as well as the role of CX_3_CR1 in directing moDC differentiation within the liver.

## 2. Materials and Methods

### 2.1. Mice and Experimental Protocol

C57BL/6 wild-type, CX_3_CR1^gfp/+^ and CX_3_CR1^gfp/gfp^ mice were housed in pathogen-free conditions and fed ad libitum with a standard chow diet and water. Liver injury was induced by injecting intra-peritoneally eight-week-old male mice with CCl_4_ (0.6 mL/kg in olive oil). Control animals received an injection with olive oil alone. Non-alcoholic streatohepatitis (NASH) was induced by feeding wild-type C57BL/6 mice with a choline/methionine-deficient diet (Laboratorio Dottori Piccioni, Gessate, Italy), as previously reported [11]. Control animals received the same diets supplemented by both choline and methionine. The CX_3_CR1 antagonist CX3-AT (European Patent EP2913060A1) [24,25] was kindly supplied by Dr. V. Julia (University of Nice, France). In some experiments, wild-type mice received an intraperitoneal injection of CX3-AT solution in sterile saline (150 µg/mice) 24 h after the administration of CCl_4_. Control animals received a similar amount of saline alone. All animals were euthanized 36 h after CCl_4_ administration. The animal experiments were performed at RWTH University Hospital Aachen and comply with ethical guidelines for animal experimentation. The protocols were approved (AZ 84-02.04.2013.A054 and AZ 84-02.04.2014.A165) by the Landesamt für Natur, Umwelt und Verbraucherschutz Nordrhein-Westfalen, Recklinghausen (Germany) according to the European legal requirements. 

### 2.2. Assessment of Liver Injury 

Livers were rapidly removed and, after rinsing in ice-cold saline, cut in pieces. Aliquots were immediately frozen in liquid nitrogen and kept at −80 °C until analysis. Two portions of each liver were fixed in 10% formalin for histology. Plasma alanine aminotransferase (ALT) and aspartate amino transferase (AST) were determined by a spectrometric kit supplied by Radim S.p.A. (Pomezia, Italy). The extension of liver injury was assessed morphologically in hematoxylin/eosin-stained liver sections.

### 2.3. Flow Cytometry Analysis and Cell Sorting of Liver Leukocytes 

Livers were digested by type-IV collagenase (Worthington, OH, USA), and intrahepatic leukocytes were isolated by multiple differential centrifugation steps according to [26]. The cell preparations were then subjected to red cell lysis by Pharmlyse (BD Biosciences, San Jose, CA, USA) and stained using combinations of the following monoclonal antibodies: CD25, I-Ab, Ly6G (BD Bioscience), CD3, CD4, CD19, CD11b, CD11c, CD45, CD80, CD40, CD88, CD26, CD35, CD105, F4-80, NK1.1, MHCII (eBioscience), CD8, CX_3_CR1, Ly6C and Ly6G (Biolegend). After surface staining, cells were fixed using 2% formalin and permeabilized using 0.5% saponin (Sigma-Aldrich, St. Louis, MO, USA). Total cell numbers were determined by adding fixed numbers of Calibrite APC beads (BD Biosciences, San Jose, CA, USA) before measurement as an internal reference [26]. Sample analysis was performed using a FACS Fortessa (BD Biosciences, San Jose, CA, USA), and data were elaborated with FlowLogic (Miltenyi Biotec, GmbH, Bergisch Gladbach, Germany) software. The gating strategy use of the identification of HDCs is shown in Supplementary Figure S1. In some experiments, CX_3_CR1^low/−^ and CX_3_CR1^high^ CD11b^+^ type-2 myeloid HDCs obtained from the livers of CCl_4_-treated mice were cell sorted using a FACS Aria-II cytometer (BD Bioscience). Sorted cells (20,000) were analyzed using the Nanostring Immunology gene array kit covering 561 genes (NanoString Technologies, Inc. Seattle, WA, USA), according to the manufacturer’s instructions. Differential gene expression was calculated using the R package DESeq2. A log2 fold change threshold greater than 2 and an adjusted *P* value of <0.01 were used for comparison.

### 2.4. mRNA Extraction and Real-Time PCR 

mRNA was extracted from snap-frozen liver fragments using the peqGOLD (peqLab, Erlangen, Germany) reagent. cDNA was generated from 1 µg of RNA using the Transcriptor first-strand cDNA synthesis kit (Roche, Basel, Switzerland). The quantitative real-time polymerase chain reaction (PCR) was performed using SYBR Green Reagent (Invitrogen, Carlsbad, CA, USA) and a QuantStudio 6 Flex Real-Time PCR System (Applied Biosystems, Carlsbad, CA, USA). All samples were run in duplicate and the relative gene expression, calculated as 2^−ΔCt^, was expressed as a fold increase over the control samples.

### 2.5. In Vitro moDC Differentiation from Bone Marrow Myeloid Cells

Myeloid cells were isolated from the tibia and femur bone marrow of CX_3_CR1^gfp/+^ and CX_3_CR1^gfp/gfp^ mice according to [27]. Red blood cells were removed with BD FACS lysing solution (BD Bioscience) and the myeloid cells were cultured for seven days in RPMI-1640 medium supplemented with 10% fetal bovine serum (FBS) with or without the addition of granulocyte-macrophage colony stimulating factor (GM-CSF; 20 ng/mL) and interleukin-4 (IL-4) (10 ng/mL). In some experiments, myeloid cells isolated from wild-type mice were cultured for seven days in 10% FBS RPMI-1640 medium in the presence of fractalkine (40 ng/mL).

### 2.6. Data Analysis and Statistical Calculations 

Statistical analyses were performed by SPSS statistical software (SPSS Inc., Chicago, IL, USA) using a one-way ANOVA test with Tukey’s correction for multiple comparisons or a Kruskal–Wallis test for non-parametric values. Significance was taken at the 5% level. Normality distribution was assessed by the Kolmogorov–Smirnov algorithm.

## 3. Results

### 3.1. Characterization of Myeloid Dendritic Cells Associated with Acute Liver Inflammation

**Figure 1:**
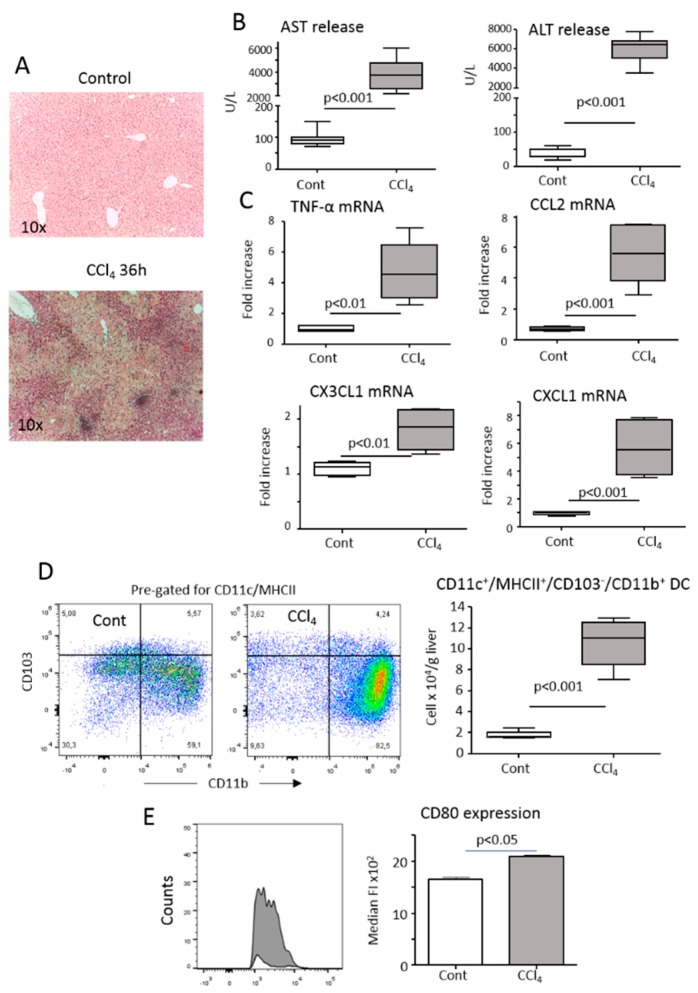
Hepatic inflammation induced by the acute administration of CCl_4_ associates with the expansion and maturation of hepatic dendritic cells (HDCs). Parenchymal damage and lobular inflammation were analyzed in wild-type mice either naïve (Cont) or 36 h after receiving an acute dose of CCl_4_ (CCl_4_). (**A**) Hematoxylin/eosin staining of formalin-fixed liver sections (magnification 10×). (**B**) Circulating levels of alanine aminotransferase (ALT) and aspartate aminotransferase (AST). (**C**) RT-PCR analysis of hepatic expression of the pro-inflammatory cyto/chemokines TNF-α, CCL2, CXCL1 and CX_3_CL1. The values are expressed as fold increase over control levels and are means ± SD of 6–8 animals in each experimental group. (**D**) The changes in the liver distribution of CD11c^+^/MHCII^high^/CD11b^+^/CD103^−^ HDCs were analyzed by flow cytometry in mice either untreated or receiving CCl_4_. (**E**) The plasma membrane expression of maturation marker CD80 was evaluated in HDCs gated for CD11b. The values are expressed as means ± SD of three different cell preparations.

According to previous observations, acute liver injury has resulted in a massive hepatic inflammatory reaction 36 h after mice poisoning with the hepatotoxic agent carbon tetrachloride (CCl_4_) (Figure 1A–C). This injury-driven inflammation was associated with an expansion of CD11c^+^/MHCII^high^/CD103^−^/CD11b^+^ myeloid HDCs (Figure 1D). Compared to healthy livers, these HDCs also underwent maturation as indicated by an increased expression of the co-stimulatory molecule CD80 (Figure 1D). 

**Figure 2:**
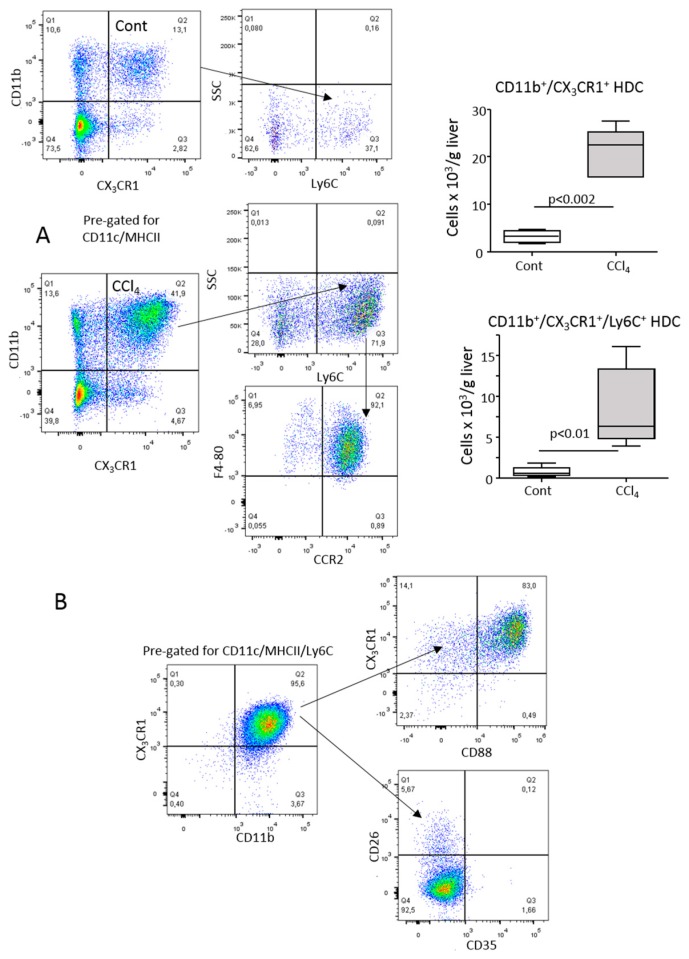
Characterization of hepatic dendritic cells (HDCs) expanding in response to acute liver injury. (**A**) CD11c^+^/MHCII^high^/CD11b^+^ HDCs from either naïve (Cont) or CCl_4_-treated mice (CCl_4_) were analyzed by flow cytometry for the expression of CX_3_CR1 and the monocyte markers Ly6C, F4-80 and CCR2. The values are expressed as means ± SD of three different cell preparations. (**B**) Relative distribution of the dendritic cell and macrophage markers CD26, CD35 and CD88 among CD11c^+^/MHCII^high^/CD11b^+^/Ly6C^+^/CX_3_CR1^+^ HDCs.

CD11b^+^ HDCs expanding in response to hepatic inflammation were characterized by a high expression of CX_3_CR1 (Figure 2A). However, they differed from CX_3_CR1^low^ type-2 myeloid HDCs present at homeostasis [4,16] by featuring the monocyte/macrophage markers Ly6C and F4-80 along with chemokine (C-C Motif) receptor 2 (CCR2), the receptor of the monocyte-recruiting chemokines CCL2/CCL7 (Figure 2A). Recently, Nakano et al. [28] reported that the combined presence of the complement C5a receptor (C5aR1 or CD88) and dipeptidyl peptidase-4 (CD26) were useful for discriminating lung-infiltrating CD11b^+^/Ly6C^+^/CD88^+^/CD26^−^ monocyte-derived dendritic cells (moDCs) from CD88^−^/CD26^+^ type-1 and type-2 myeloid dendritic cells. In our hands, CD11b^+^/Ly6C^+^/CX_3_CR1^+^ HDCs detectable in the livers of CCl_4_-treated mice were largely CD88^+^ and did not express CD26 (Figure 2B). These cells were also negative for the C3b-complement receptor (CD35) (Figure 2B), which is common on hepatic macrophages. Altogether, these data suggested a tentative identification of CD11b^+^/Ly6C^+^/CD88^+^/CX_3_CR1^+^ HDCs as moDCs. In line with the pro-inflammatory features of moDCs [21,22,23], a nanostring gene array comparing CX_3_CR1^low/−^ and CX_3_CR1^high^/CD11b^+^ myeloid HDCs obtained from CCl_4_-treated mice revealed that among the 75 genes upregulated in the CX_3_CR1^high^ subset were those for interleukin-1β (IL-1β), toll-like receptors (Tlr-1,2,4,8), chemokines (CCL-2,3,4,6,7,9,12), immunoglobulin Fc receptors (CD16-2, CD32, CD64), CD14, macrophage scavenger receptor 1, urokinase and urokinase receptor (Supplementary Table S1). Interestingly, these cells also showed an enhanced gene expression of anti-inflammatory mediators such as interleukin-1 receptor antagonist (IL-1a) and transforming growth factor β-1 (TGFβ-1) (Supplementary Table S1). 

**Figure 3:**
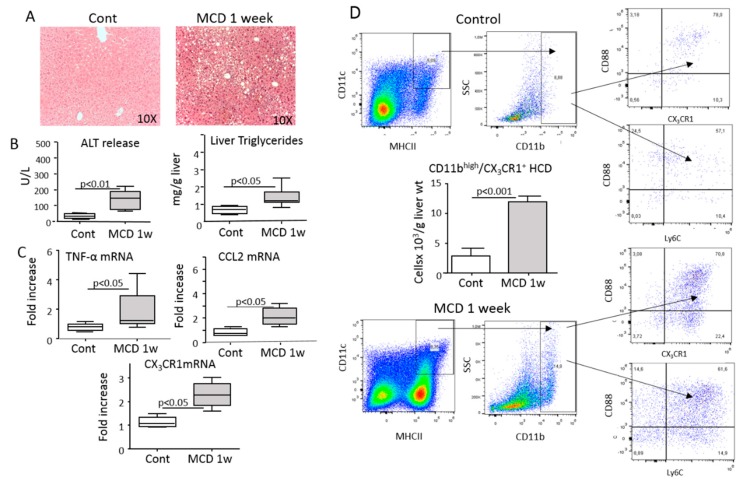
Monocyte-derived dendritic cells (moDCs) account for HDC expansion associated with the onset of non-alcoholic steatohepatitis (NASH). Steatohepatitis was induced by feeding wild-type mice with a choline/methionine-deficient (MCD) diet for one week. (**A**) Hematoxylin/eosin staining of formalin-fixed liver sections (magnification 10×). (**B**) Circulating levels of alanine aminotransferase (ALT) and liver content of triglycerides. (**C**) RT-PCR analysis of the hepatic expression of the pro-inflammatory cyto/chemokines TNF-α, CCL2. Values are expressed as means ± SD of 5–6 animals in each experimental group. (**D**) Flow cytometry analysis of the changes in the hepatic distribution of CD11c^+^/MHCII^high^/CD11b^high^/CD88^+^/Ly6c^+^ moDCs analyzed in mice either untreated (Cont) or receiving the an MCD diet. Values are expressed as means ± SD of three different cell preparations.

Myeloid HDC expansion has been observed in liver reperfusion injury as well as non-alcoholic steatohepatitis (NASH) induced by feeding mice with a methionine/choline-deficient (MCD) diet [10,14]. Since we previously reported the involvement of *CX_3_CR1*-expressing HDCs in the progression of NASH [11], we investigated whether these cells might have features of moDCs. Figure 3 shows that the onset of NASH in mice fed for one week with the MCD diet was characterized by an upregulation in liver CX3CR1 transcript and by an increase in CD11b^+^ myeloid HDCs that co-expressed CX_3_CR1, CD88 and Ly6C, suggesting that moDCs might also account for HDC activation associated with steatohepatitis. 

### 3.2. Interference with CX_3_CR1 Affects Monocyte Maturation to Dendritic Cells in Injured Livers 

**Figure 4:**
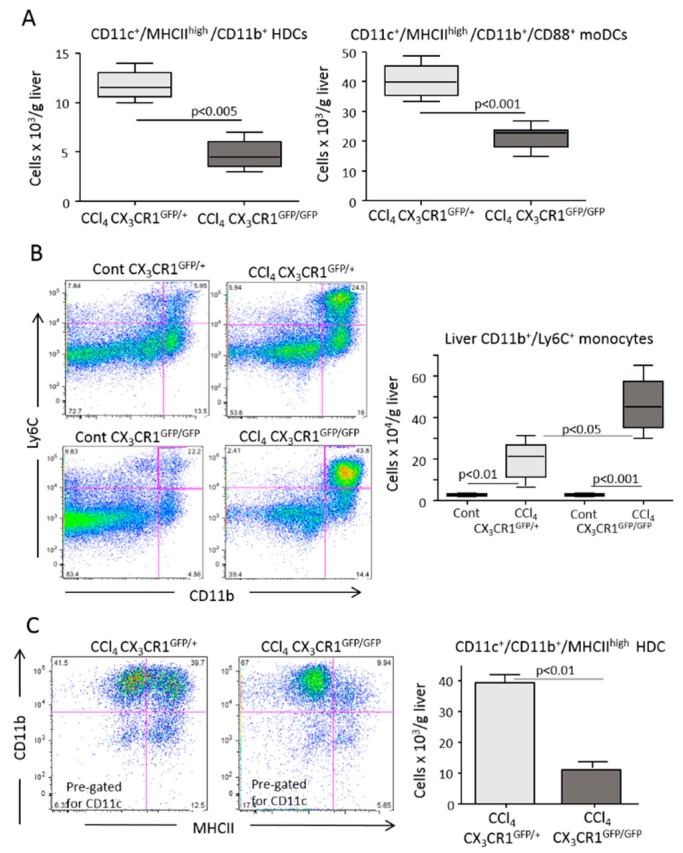
The lack of CX_3_CR1 reduced the differentiation of monocyte-derived dendritic cells (moDCs) in response to liver inflammation. The liver distribution of CD11b^+^ myeloid dendritic cells, moDCs and monocytes were analyzed by flow cytometry in the livers of CX_3_CR1^gfp/+^ and CX_3_CR1^gfp/gfp^ mice 36 h after receiving an acute dose of CCl_4_. (**A**) The prevalence of CD11c^+^/MHCII^high^/CD11b^+^ myeloid dendritic cells and CD11c^+^/MHCII^high^/CD11b^+^/CD88^+^ moDCs in CX_3_CR1^gfp/+^ and CX_3_CR1^gfp/gfp^ mice receiving CCl_4_. (**B**) Hepatic distribution of Ly6G^−^/CD11b^high^/Ly6C^high^ monocytes in control and CCl_4_-treated CX_3_CR1^gfp/+^ and CX_3_CR1^gfp/gfp^ mice. (**C**) Impaired expression of MHCII by CD11c^+^/CD11b^+^ myeloid cells in CCl_4_-treated CX_3_CR1^gfp/+^ and CX_3_CR1^gfp/gfp^ mice. The values are expressed as means ± SD of three different cell preparations or 5–6 animals.

Following the observation that fractalkine (CX_3_CL1) was upregulated during hepatic inflammation in parallel with the expansion of CX_3_CR1^+^ moDCs (Figure 1), we addressed the possible role of CX_3_CR1-mediated signaling in modulating hepatic moDC responses. Experiments performed on CX_3_CR1-deficient homozygous mice involved having a green fluorescent protein (gfp) inserted in the CX_3_CR1 gene (CX_3_CR1^gfp/gfp^), showing that the expansion of myeloid dendritic cells associated with CCl_4_ poisoning were strongly reduced in CX_3_CR1^gfp/gfp^ mice compared with CX_3_CR1-proficient (CX_3_CR1^+/gfp^) animals. Such an effect specifically involved a fraction of liver CD11c^+^/MHCII^high^/CX_3_CR1^+^/CD11b^+^/CD88^+^ moDCs (Figure 4A and Supplementary Figure S2). Conversely, no significant differences were appreciable between CCl_4_-treated CX_3_CR1^gfp/gfp^ and CX_3_CR1^+/gfp^ mice in the hepatic distribution of CX_3_CR1^+^/F4-80^+^/CD11b^high^ monocytes/macrophages (Supplementary Figure S3). In a similar manner, the lack of CX_3_CR1 did not affect the liver recruitment of Ly6G^−^/CD11b^high^/Ly6C^high^ monocytes that were instead more prevalent in CX_3_CR1^gfp/gfp^ mice (Figure 4B). However, we observed that in the absence of CX_3_CR1 cells that were CD11b^+^/Ly6C^+^ and positive for the common dendritic cell marker CD11c^+^ failed to fully express MHCII (Figure 4C), suggesting a possible role of CX_3_CR1 signaling in the differentiation of monocytes from moDCs.

**Figure 5:**
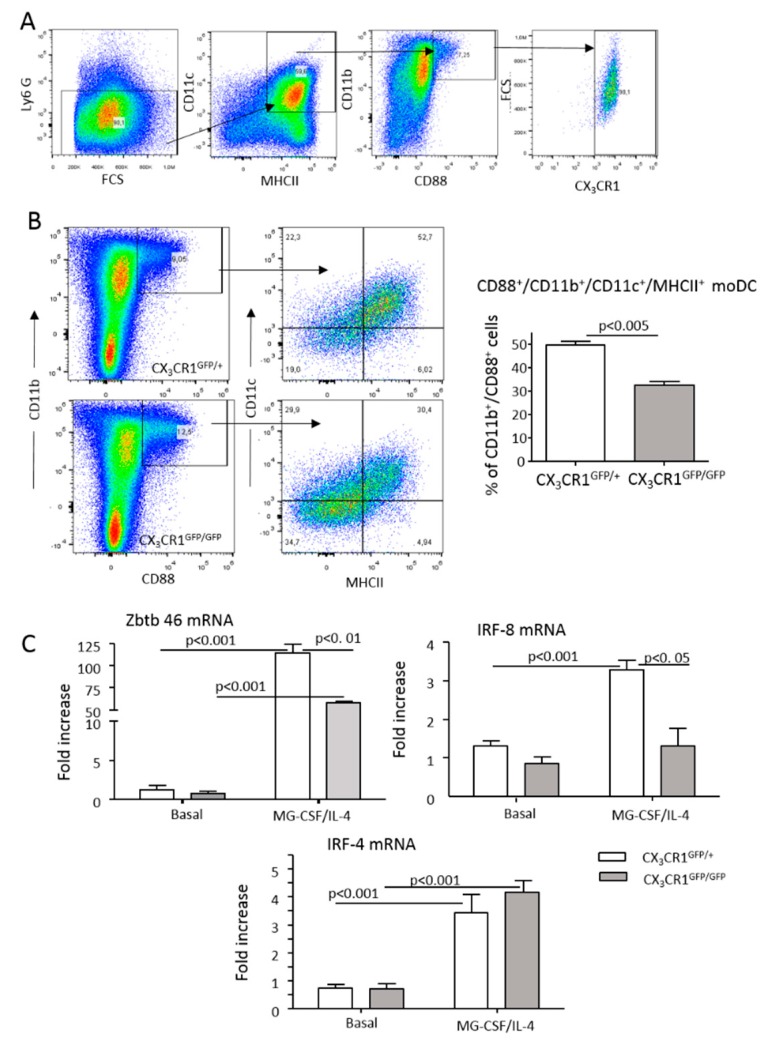
A lack of CX_3_CR1 affects the in vitro differentiation of monocyte-derived dendritic cells (moDCs). MoDCs were obtained in vitro by a seven-day culture of bone marrow myeloid cells from either CX_3_CR1^gfp/+^ or CX_3_CR1^gfp/gfp^ mice with granulocyte-macrophage colony stimulating factor (GM-CSF) and interleukin-4 (IL-4). (**A**) CX_3_CR1 expression in Ly6G^−^/CD11b^+^/CD88^+^/CD11c^+^/MHCII^high^ moDC originating from GM-CF/IL-4-treated bone marrow myeloid cells. (**B**) The effect of CX_3_CR1 on the in vitro differentiation of CD11b^+^/CD88^+^/CD11c^+^/MHCII^high^ moDCs. (**C**) Effect of CX_3_CR1 on the expression of the transcription factors Zbtb46, IRF-4 and IRF-8 implicated in moDC differentiation. The values are expressed as means ± SD of 3–5 different cell preparations.

MoDCs can be readily obtained in vitro by culturing bone marrow myeloid cells with granulocyte-macrophage colony stimulating factor (GM-CSF) and interleukin-4 (IL-4) [29]. We observed that Ly6G^−^/CD11b^+^/CD88^+^/CD11c^+^/MHCII^high^ moDC originating from GM-CSF/IL-4-treated bone marrow myeloid cells expressed CX_3_CR1 (Figure 5A). Thus, we used these cells to further investigate the role of CX_3_CR1 in moDC differentiation. As shown in Figure 5B, the fraction of moDCs maturating from the bone marrow of CX_3_CR1^gfp/gfp^ mice was 35% lower than that from CX_3_CR1^+/gfp^ animals. The absence of CX_3_CR1 also influenced the spontaneous differentiation of moDCs when bone marrow myeloid cells were cultured seven days in calf serum-supplemented medium without GM-CSF/IL-4 (24 ± 2.1% vs 13 ± 2.1%; *p* < 0.01). In this setting, the lack of CX_3_CR1 specifically hampered MHCII expression via CD11b^+^/CD11c^+^ pre-dendritic cells (Supplementary Figure S4). Conversely, the addition of fractalkine (40 ng/mL) to the medium promoted moDC differentiation (28 ± 0.6% vs 32 ± 1.2%; *p* < 0.05), indicating a CX_3_CR1 action in the processes leading to full moDC development. To further evaluate such a possibility, we investigated CX_3_CR1 effects on the mRNA levels of the transcription factors Zbtb46, interferon responsive factor-4 (IRF-4) and interferon responsive factor-8 (IRF-8) that have been implicated in moDC maturation induced by GM-CSF/IL-4 [29,30,31,32]. All three transcription factors were significantly upregulated in the cells exposed to GM-CSF/IL-4 (Figure 4C). The lack of CX_3_CR1 significantly reduced Zbtb46 and IRF-8 mRNAs, while IRF-4 mRNA was unaffected (Figure 5C), further pointing to a role of CX_3_CR1 in monocyte differentiation to moDCs. 

### 3.3. Interference with CX_3_CR1-Mediated moDC Differentiation Ameliorates Acute Liver Injury and Inflammation

Several studies have implicated moDCs in sustaining tissue injury and inflammation in different tissues [21,22]. Unfortunately, since the lack of CX_3_CR1 affects the anti-inflammatory properties of resident CX_3_CR1^+^ type-2 myeloid HDCs [16], CX_3_CR1^gfp/gfp^ mice were unsuitable to address the possible role of CX_3_CR1^+^ moDCs in sustaining inflammatory responses associated to hepatic damage. To overcome such a limitation, we took advantage of CX3-AT, a NH2-terminal CX_3_CL1-derived peptide that has been previously reported to block CX_3_CR1 signaling in lymphocytes [24,25]. For these experiments, mice were treated with a single dose of CX3-AT (150 µg in saline i.p.) 24 h after the administration of CCl_4_, and the effects on hepatic inflammation were monitored over the following 12 h. At this dosage, CX3-AT was not hepatotoxic and did not affect the liver distribution of monocyte/macrophages and CX_3_CR1^+^ type-2 myeloid HDCs (Supplementary Figure S5). 

**Figure 6:**
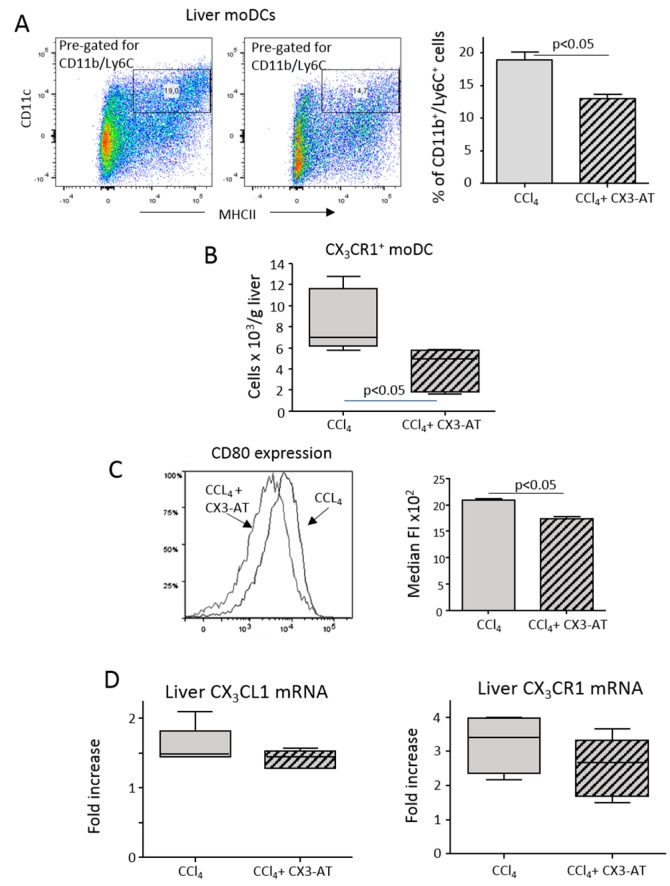
The CX_3_CL1 antagonist CX3-AT reduces the expansion of monocyte-derived dendritic cells (moDCs) in response to hepatic injury. Liver dendritic cells were analyzed by flow cytometry in mice receiving CCl_4_ alone or in combination with CX3-AT. (**A**,**B**) Liver distribution of CD11b^+^/Ly6C^+^ moDCs and CX_3_CR1-expressing moDCs. (**C**) Plasma membrane expression of the maturation marker CD80 in moDCs for CCl_4_-treated mice receiving or not receiving CX3-AT. The values are expressed as means ± SD of three different cell preparations. (**D**) RT-PCR analysis of the hepatic transcripts for CX_3_CL1 and CX_3_CR1. The values are expressed as fold increase over control levels and are means ± SD of 6–8 animals in each experimental group.

In line with the data obtained using CX_3_CR1-deficient mice, CX3-AT treatment affected moDC expansion in response to CCl_4_-induced liver injury (Figure 6A), specifically reducing the fraction of CX_3_CR1^+^ moDCs (Figure 6B). We also observed that CX3-AT addition lowered moDC expression of the maturation marker CD80 (Figure 6C) without interfering with the hepatic expression of CX_3_CL1 and CX_3_CR1 (Figure 6D). Similarly, no appreciable changes were observed in the liver distribution of T-lymphocytes, natural killer (NK) and natural killer T cells (NKT) cells that also rely on CX_3_CR1 signaling (Supplementary Figure S6). 

**Figure 7:**
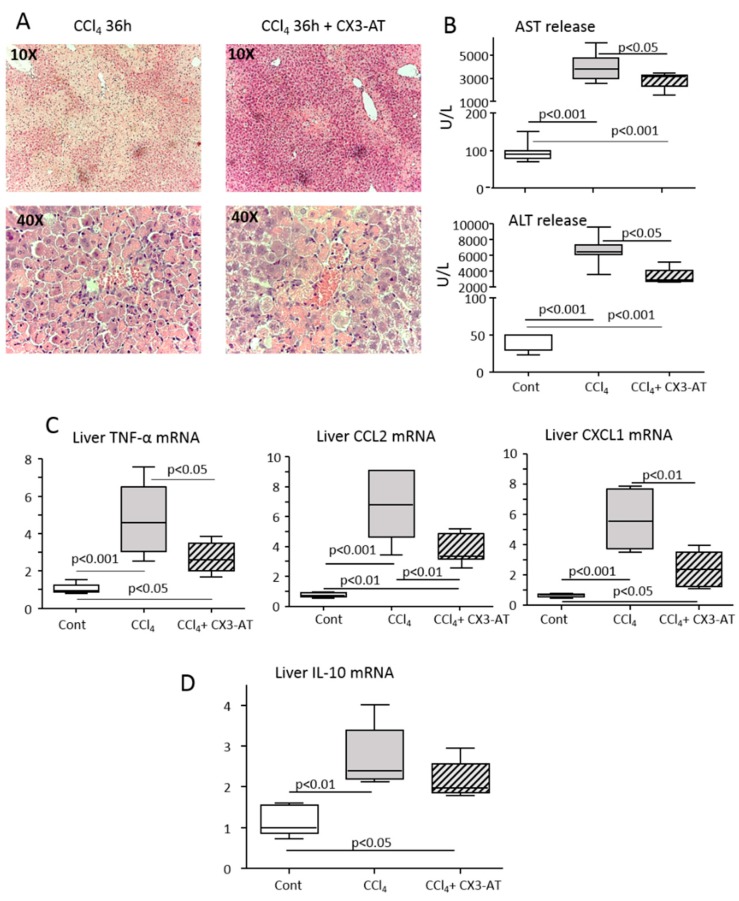
The CX_3_CL1 antagonist CX3-AT improves liver injury and inflammation in mice receiving CCl_4_. Parenchymal damage and lobular inflammation were analyzed in mice receiving CCl_4_ alone and in combination with CX3-AT. (**A**) Hematoxilin/eosin staining of formalin-fixed liver sections (magnification 10× and 40×). (**B**) Circulating levels of alanine aminotransferase (ALT) and aspartate aminotransferase (AST). (**C**,**D**) RT-PCR analysis of the hepatic expression of pro-inflammatory cyto/chemokines TNF-α, CCL2 and CXCL1, as well as IL-10. The values are expressed as fold increase over control levels and are means ± SD of 6–8 animals in each experimental group.

The animals receiving CX3-AT liver histology showed a significant reduction of parenchymal necrosis and inflammatory infiltrates (Figure 7A). Consistently, CX3-AT treatment ameliorated transaminase release (Figure 7B) and reduced the hepatic expression of the pro-inflammatory cyto/chemokines TNF-α, CCL2 and CXCL1 (Figure 7C). We have previously reported that during homeostasis type-2 myeloid HDCs expressing the fractalkine receptor CX_3_CR1 and producing IL-10 are detectable in mice livers and counteract acute hepatic inflammation [16]. Figure 7D shows that CX3-AT did not interfere with these cells, as the upregulation in hepatic IL-10 transcript was unaffected. Altogether, these data indicate that CX_3_CR1-dependent moDCs effectively contribute to hepatic inflammation in response to liver injury.

## 4. Discussion

Growing evidence indicates that HDCs play an important role in modulating hepatic immune and inflammatory responses both at homeostasis and in liver diseases [4,6]. Along with that, experiments using different models of acute and chronic liver injury have provided evidence that myeloid HDCs expand and mature in response to inflammatory stimuli sustaining the evolution of hepatic damage [9,10,33]. However, the specific features of these HDCs have not been investigated in detail. Our present data add to the role of HDCs in liver pathology by showing that HDC expansion in response to inflammatory stimuli involves cells featuring CD11b as type-2 classical HDCs along with the fractalkine receptor CX_3_CR1. The presence of CX_3_CR1 in HDCs has been previously documented in the liver at homeostasis [4,16]. However, CX_3_CR1^high^ HDCs in inflamed livers differ from CX_3_CR1^low^ HDCs in naïve mice because they express the monocyte markers F4-80 and CCR2 and are Ly6C^high^. Furthermore, the former are also characterized by the presence of the complement C5a receptor (C5aR1 or CD88), while they are negative for the dipeptidyl peptidase-4 (CD26), two surface markers that discriminate CD11b^+^ monocyte-derived DCs (CD88^+^/CD26^−^) from type-1 and type-2 classical DCs (CD88^−^/CD26^+^) [28]. In line with this, Ly6C^+^/CX_3_CR1^+^/CD88^+^ moDCs also account for HDC expansion occurring at the onset of experimental NASH, confirming the previous implication of moDC in sustaining liver injury and inflammation in experimental models of chronic steatohepatitis [11]. Furthermore, Huang et al. [34] have reported that CD11b^+^ moDCs contribute to the formation of intrahepatic myeloid-cell aggregates for T-cell expansion (iMATEs), which are responsible for CD8^+^ T-lymphocyte stimulation during viral infection. On these bases, we propose that the differentiation of dendritic cells from liver-infiltrating monocytes might represent a mechanism to rapidly expand the pool of HDCs in response to pro-inflammatory stimuli. Nonetheless, we are well aware that the above surface markers do not allow a definitive differentiation of moDCs from liver-infiltrating inflammatory monocyte-derived macrophages that are also Ly6C^high^/CX_3_CR1^+^ and can express CD11c, MHCII and CD88 to varying extents [3,35].

The experiment using CX_3_CR1-deficient mice showed that the expansion of moDCs in response to liver injury requires CX_3_CR1. It is noteworthy that, despite CX_3_CR1 being widely present in liver myeloid cells (including inflammatory monocyte-derived macrophages), such an effect is specific to moDCs. In fact, in line with a previous report [36], the lack of CX_3_CR1 in CCl_4_-treated mice did not affect the pool of liver-infiltrating monocyte-derived macrophages, further indicating that CX_3_CR1^+^ moDCs might represent a different cell subset. At present, CX_3_CR1 function in moDCs is still poorly characterized. David et al. have shown that CX_3_CR1 contributes to liver dendritic cell replenishment after selective depletion by directing bone marrow-derived precursors [4]. A recent report has also shown that CX_3_CR1/CX_3_CL1 interaction is critical for monocyte adhesion to endothelial cells and their migration into atherosclerotic plaques [37]. However, the effects observed in CX_3_CR1-deficient mice receiving CCl_4_ do not involve the hepatic recruitment of monocytes. This is consistent with the recent observation that chemokine (C-C Motif) receptor 2 (CCR2) is the main responsible for monocyte attraction within injured livers [38]. On the other hand, we have observed that CX_3_CR1 deficiency impairs the capacity of dendritic cell precursors to fully express *MHCII*. Moreover, lack of CX_3_CR1 reduces in vitro moDC maturation from bone marrow myeloid cells incubated with MG-CSF/IL-4. Altogether these data suggest that CX_3_CR1-mediated signals are required for moDC differentiation. Supporting this view, we have observed that in MG-CSF/IL-4-treated myeloid cells CX_3_CR1 influences the expression of Zbtb46 and IRF-8, two transcription factors implicated in driving monocyte differentiation to moDC [30,31]. However, we cannot exclude that CX_3_CR1 might have additional effects on moDCs, as this receptor is required for the survival of liver-infiltrating monocyte-derived macrophages [36].

It is well established that moDCs are not only effective in antigen presentation to lymphocytes, but also produce pro-inflammatory mediators contributing to sustaining inflammatory processes [21,22,23]. Regarding this latter finding we have observed that interfering with liver CX_3_CR1^+^ moDC differentiation using the CX_3_CR1-blocker CX3-AT ameliorates lobular inflammation and parenchymal damage following acute CCl_4_ poisoning. These findings are consistent with previous data showing that the H_2_S donor NaHS lowers hepatic TNFα levels and ameliorates parenchymal injury in an experimental model of chronic steatohepatitis by selectively affecting the development of CX_3_CR1-expressing HDCs [11]. Taken together these observations indicate that CX_3_CR1^+^ moDCs can contribute to sustaining hepatic injury and inflammation through the production of pro-inflammatory mediators. Such an interpretation is in line with the data of Connolly et al. [9] showing that TNFα-producing HDCs drive hepatic inflammation in mice via thioacetamide-induced fibrosis.

The results of this work also provide evidence that CX_3_CR1-expressing HDCs might have different functional capabilities in relation to their origins and/or exposure to different environmental stimuli. In fact, during homeostasis liver CX_3_CR1^+^/CD11b^+^ type-2 myeloid HDCs produce IL-10 and have anti-inflammatory capabilities [16], thereby contributing to hepatic tolerogenic environment. Conversely, in injured livers the differentiation of monocytes into CX_3_CR1^+^ moDCs, in response to inflammatory stimuli, is responsible for the development of HDCs with pro-inflammatory and immune-stimulating activities. These opposite functions of dendritic cells expressing similar surface markers might help to explain the inconsistent results so far obtained in assessing the role of HDCs in the evolution of chronic liver diseases [12,13,14,15,16].

## 5. Conclusions

In conclusion, the present results add functional data regarding the complex role of dendritic cells in the mechanisms of liver injury, indicating that the rapid expansion of HCDs in response to hepatic injury involve monocyte differentiation to inflammatory moDCs. Furthermore, our data point to the importance of CX_3_CR1/CX_3_CL1 dyad in modulating moDC differentiation within the liver.

## Supplementary Materials

The following are available online at https://www.mdpi.com/2073-4409/8/9/1099/s1: Supplementary Table S1 Changes in gene expression between CX_3_CR1^low/−^ and CX_3_CR1^high^ CD11b^+^ myeloid hepatic dendritic cells (HDCs) from CCl_4_-treated mice; Supplementary Figure S1 Gating strategy for liver dendritic cells; Figure S2 The lack of CX_3_CR1 affects the pool of monocyte-derived dendritic cells (moDCs); Figure S3 The lack of CX_3_CR1 did not interfere with the liver recruitment of CX_3_CR1^+^ monocytes/macrophages; Figure S4 The lack of CX_3_CR1 affects in vitro differentiation of monocyte-derived dendritic cells (moDCs); Figure S5 Effect of CX_3_CR1 antagonist CX3-AT in naïve mice; Figure S6 CX3-AT does not affect the liver distribution of different lymphocyte sub-sets during hepatic inflammation.
