# Supplementary material for: CX3CR1 Mediates the Development of Monocyte-Derived Dendritic Cells during Hepatic Inflammation
Source: Cells. 2019 Sep 18;8(9):1099. doi: 10.3390/cells8091099 (PMC6770190; doi:10.3390/cells8091099)
Supplement: Supplementary file 1 [file cells-08-01099-s001.pdf]

CX3CR1 MEDIATES THE DEVELOPMENT OF MONOCYTE-DERIVED DENDRITIC CELLS DURING HEPATIC INFLAMMATION.

Supplementary material

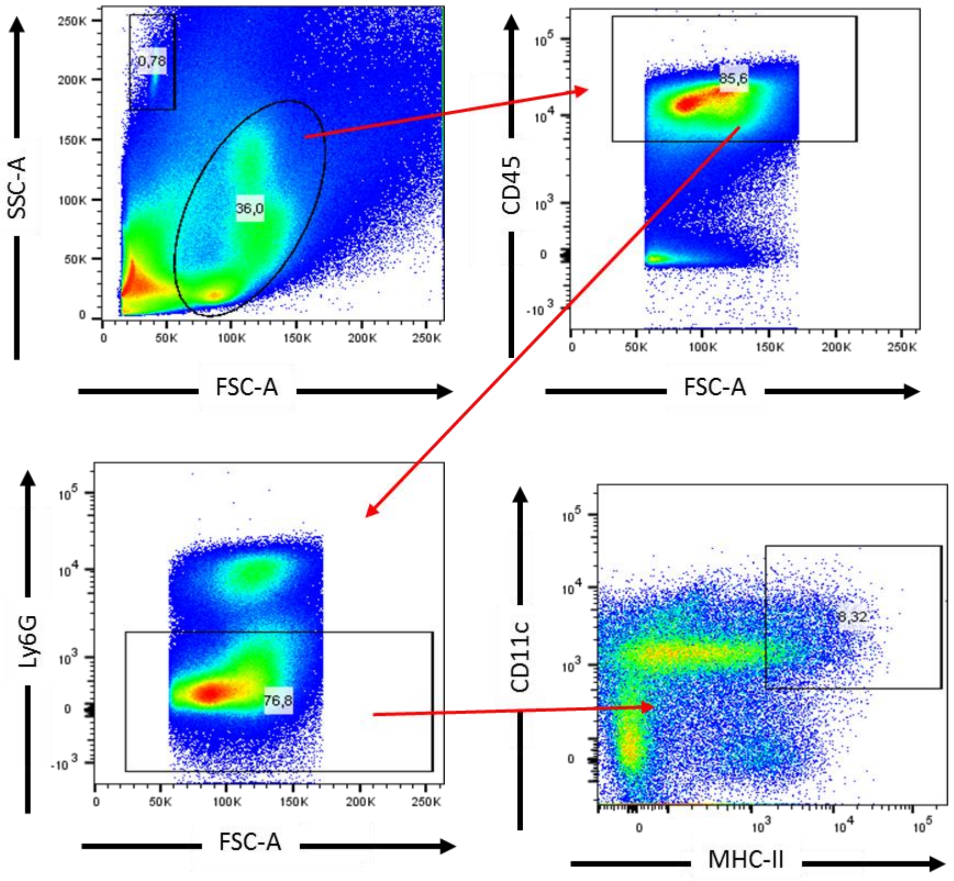

Supplementary Figure 1: Liver CD45^{+} myeloid cells were pre-gated for Ly6G negative cells for excluding granulocytes and HDCs subsequently analyzed among the cells that were CD11c^{+} and had high expression of MHCII.

Supplementary Table 1

Changes in gene expression between $CX_3CR1^{low/-}$ and CX_3CR1^{high} $CD11b^{+}$ myeloid hepatic dendritic cells (HDCs) from CCl_4-treated mice

Genes up-regulated in CX_3CR1^{high} HDCs			
Gene	Fold changes	P value	Full name
App	4,01702	5,89E-05	amyloid beta (A4) precursor protein
C1qa	9,75881	1,69E-22	complement component 1, q subcomponent, alpha polypeptide
C1qb	9,19882	3,62E-20	complement component 1, q subcomponent, beta polypeptide
Ccl12	2,51899	0,011769	chemokine (C-C motif) ligand 12
Ccl2	6,53486	6,37E-11	chemokine (C-C motif) ligand 2
Ccl3	4,99649	5,84E-07	chemokine (C-C motif) ligand 3
Ccl4	4,42552	9,62E-06	chemokine (C-C motif) ligand 4
Ccl6	3,9311	8,46E-05	chemokine (C-C motif) ligand 6
Ccl7	2,60184	0,009272	chemokine (C-C motif) ligand 7
Ccl9	4,17294	3,01E-05	chemokine (C-C motif) ligand 9
Ccr2	3,35195	0,000802	chemokine (C-C motif) receptor 2
Ccr5	3,23358	0,001222	chemokine (C-C motif) receptor 5
Cd14	6,13325	8,61E-10	CD14 antigen
Cd36	2,94367	0,003243	CD36 antigen
Cd44	4,89958	9,60E-07	CD44 antigen
Cd81	6,49623	8,24E-11	CD81 antigen
Cd9	3,06253	0,002195	CD9 antigen
Cdkn1a	4,65279	3,27E-06	cyclin-dependent kinase inhibitor 1A (P21)
Cebpb	6,6083	3,89E-11	CCAAT/enhancer binding protein (C/EBP), beta
Cfb	2,10895	0,034949	complement factor B
Cmklr1	6,72268	1,78E-11	chemokine-like receptor 1
Csf1r	6,36466	1,96E-10	colony stimulating factor 1 receptor
Csf3r	3,84985	0,000118	colony stimulating factor 3 receptor (granulocyte)
Ctsc	5,89204	3,81E-09	cathepsin C
Ctss	3,2198	0,001283	cathepsin S
Cx3cr1	13,1318	2,16E-39	chemokine (C-X3-C) receptor 1
Cybb	4,92213	8,56E-07	cytochrome b-245, beta polypeptide
Ddx58	2,66239	0,007759	DEAD (Asp-Glu-Ala-Asp) box polypeptide 58
Emr1	9,69028	3,32E-22	EGF-like module containing, mucin-like, hormone receptor-like sequence 1
Fcer1g	4,20145	2,65E-05	Fc receptor, IgE, high affinity I, gamma polypeptide
Fcgr1	9,18398	4,15E-20	Fc receptor, IgG, high affinity I
Fcgr2b	4,65036	3,31E-06	Fc receptor, IgG, low affinity IIb
Fcgr4	7,62716	2,40E-14	Fc receptor, IgG, low affinity IV
Fn1	5,74254	9,33E-09	fibronectin 1
Ifi204	5,51675	3,45E-08	interferon activated gene 204
Ifnar2	4,27785	1,89E-05	interferon (alpha and beta) receptor 2
Ifngr2	2,48297	0,013029	interferon gamma receptor 2
Ikbke	5,38026	7,44E-08	inhibitor of kappaB kinase epsilon
Il10rb	4,22073	2,44E-05	interleukin 10 receptor, beta
Il1b	2,70627	0,006804	interleukin 1 beta
Il1rn	3,33231	0,000861	interleukin 1 receptor antagonist
Irf7	3,6732	0,00024	interferon regulatory factor 7
Itga6	4,92143	8,59E-07	integrin alpha 6
Itgal	5,4946	3,92E-08	integrin alpha L
Lair1	5,00721	5,52E-07	leukocyte-associated Ig-like receptor 1
Lilra5	3,17543	0,001496	leukocyte immunoglobulin-like receptor, subfamily A (with TM domain), member 5
Litaf	2,82839	0,004678	LPS-induced TN factor
Ly86	2,79276	0,005226	lymphocyte antigen 86
Maf	4,71703	2,39E-06	avian musculoaponeurotic fibrosarcoma (v-maf) AS42 oncogene homolog
Msr1	8,39109	4,82E-17	macrophage scavenger receptor 1
Ncf4	3,33874	0,000842	neutrophil cytosolic factor 4
Nfil3	3,31981	0,000901	nuclear factor, interleukin 3, regulated
Nfkbiz	2,76339	0,00572	nuclear factor of kappa light polypeptide gene enhancer in B cells inhibitor, zeta
Npc1	2,9948	0,002746	Niemann Pick type C1
Pdgfb	3,85957	0,000114	platelet derived growth factor, B polypeptide
Plau	6,42638	1,31E-10	plasminogen activator, urokinase
Plaur	4,15787	3,21E-05	plasminogen activator, urokinase receptor
Pparg	4,70638	2,52E-06	peroxisome proliferator activated receptor gamma
Prdm1	2,975	0,00293	PR domain containing 1, with ZNF domain
Ptafr	2,89613	0,003778	platelet-activating factor receptor
Ski	2,58243	0,009811	ski sarcoma viral oncogene homolog (avian)

Smad3	2,70074	0,006918	MAD homolog 3 (Drosophila)
Stat2	2,51455	0,011919	signal transducer and activator of transcription 2
Tgfb1	3,16093	0,001573	transforming growth factor, beta 1
Tgfbi	5,15178	2,58E-07	transforming growth factor, beta induced
Tgfbr1	5,45649	4,86E-08	transforming growth factor, beta receptor I
Tlr1	4,84139	1,29E-06	toll-like receptor 1
Tlr2	2,69617	0,007014	toll-like receptor 2
Tlr4	3,61259	0,000303	toll-like receptor 4
Tlr8	6,27586	3,48E-10	toll-like receptor 8
Tnfrsf11a	3,04365	0,002337	tumor necrosis factor receptor superfamily, member 11a
Tnfrsf14	3,13939	0,001693	tumor necrosis factor receptor superfamily, member 14 (herpesvirus entry mediator)
Tnfrsf1b	4,96437	6,89E-07	tumor necrosis factor receptor superfamily, member 1b
Tnfsf12	2,79525	0,005186	tumor necrosis factor (ligand) superfamily, member 12
Trem2	5,50484	3,70E-08	triggering receptor expressed on myeloid cells 2
Tyrobp	2,66403	0,007721	TYRO protein tyrosine kinase binding protein

Genes down-regulated in CX_3CR1^{high} HDCs

Gene	Fold changes	P value	Full name
Ahr	-2,611174	0,009023	aryl-hydrocarbon receptor
Btla	-6,957308	3,47E-12	B and T lymphocyte associated
Ccl22	-4,362738	1,28E-05	chemokine (C-C motif) ligand 22
Ccr7	-7,241423	4,44E-13	chemokine (C-C motif) receptor 7
Cd244	-2,605379	0,009177	CD244 natural killer cell receptor 2B4
Cd24a	-5,198278	2,01E-07	CD24a antigen
Cd34	-5,099496	3,41E-07	CD34 antigen
Cd7	-5,286396	1,25E-07	CD7 antigen
Cd82	-2,956234	0,003114	CD82 antigen
Cd83	-3,163861	0,001557	CD83 antigen
Ciita	-5,820575	5,86E-09	class II transactivator
Dpp4	-5,877618	4,16E-09	dipeptidylpeptidase 4
Fyn	-3,696658	0,000218	Fyn proto-oncogene
Gpr183	-2,784596	0,005359	G protein-coupled receptor 183
H2-Aa	-2,968497	0,002993	histocompatibility 2, class II antigen A, alpha
H2-DMb2	-3,67274	0,00024	histocompatibility 2, class II, locus Mb2
H2-Ob	-5,529531	3,21E-08	histocompatibility 2, O region beta locus
Icosl	-4,664422	3,09E-06	icos ligand
Ifitm1	-8,456888	2,75E-17	interferon induced transmembrane protein 1
Il1r2	-6,064631	1,32E-09	interleukin 1 receptor, type II
Il7r	-2,692397	0,007094	interleukin 7 receptor
Itgax	-2,68947	0,007157	integrin alpha X
Jak2	-6,927284	4,29E-12	Janus kinase 2
Kit	-4,144838	3,40E-05	kit oncogene
Ltb	-2,913954	0,003569	lymphotoxin B
Ltb4r1	-2,481954	0,013066	leukotriene B4 receptor 1
Relb	-5,286109	1,25E-07	avian reticuloendotheliosis viral (v-rel) oncogene related B
Runx3	-4,727032	2,28E-06	runt related transcription factor 3
S100a8	-4,289814	1,79E-05	S100 calcium binding protein A8 (calgranulin A)
Sell	-4,53454	5,77E-06	selectin, lymphocyte
Sigirr	-3,943093	8,04E-05	single immunoglobulin and toll-interleukin 1 receptor (TIR) domain
Slamf7	-3,795066	0,000148	SLAM family member 7
Stat5a	-3,201358	0,001368	signal transducer and activator of transcription 5A
Tagap	-3,1848	0,001449	T cell activation Rho GTPase activating protein
Tbx21	-2,340494	0,019258	T-box 21
Tcf7	-2,9563	0,003114	transcription factor 7, T cell specific
Tlr3	-4,154069	3,27E-05	toll-like receptor 3
Traf1	-6,474935	9,49E-11	TNF receptor-associated factor 1
Xcr1	4,51824	6,24E-06	chemokine (C motif) receptor 1
Zeb1	4,141243	3,45E-05	zinc finger E-box binding homeobox 1

$CX_3CR1^{low/-}$ and $CX_3CR1^{high}/CD11b^+$ myeloid HDCs were obtained from the livers of CCl_4-treated mice and were cell sorted using a FACS Aria cytometer. Gene expression was analysed using Nanostring gene array.

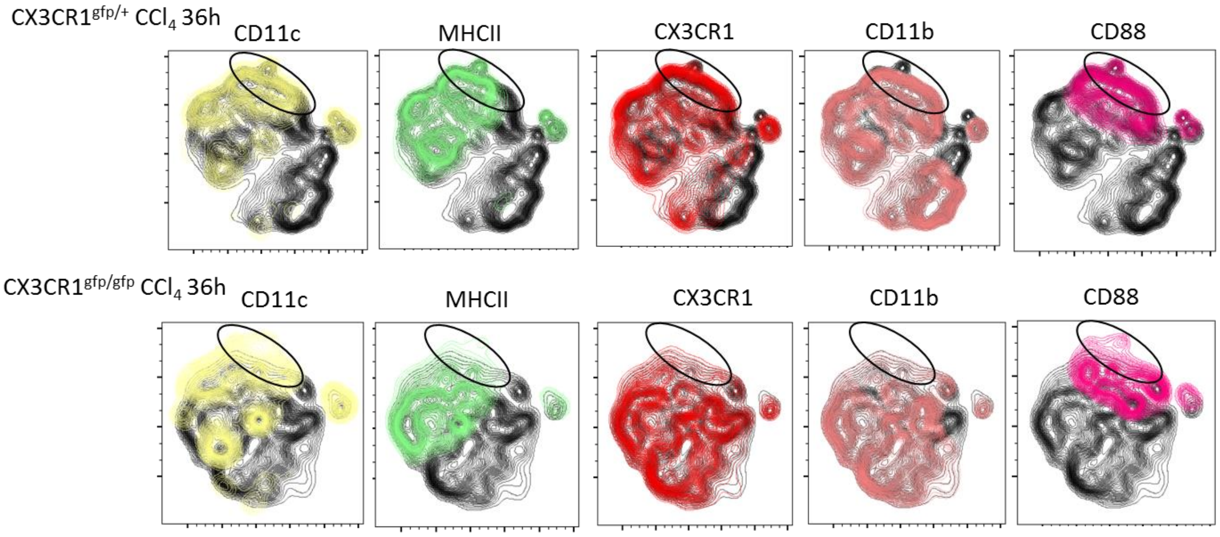

Supplementary Figure 2: The lack of CX_3CR1 affects the pool of monocyte-derived dendritic cells (moDCs).

Flow cytometry plot showing the cluster of moDCs identified as co-expressing CD11c, MHCII, CX_3CR1, CD11b and CD88 in the livers of $CX_3CR1^{gfp/+}$ and $CX_3CR1^{gfp/gfp}$ mice 36 hours after receiving an acute dose of CCl_4.

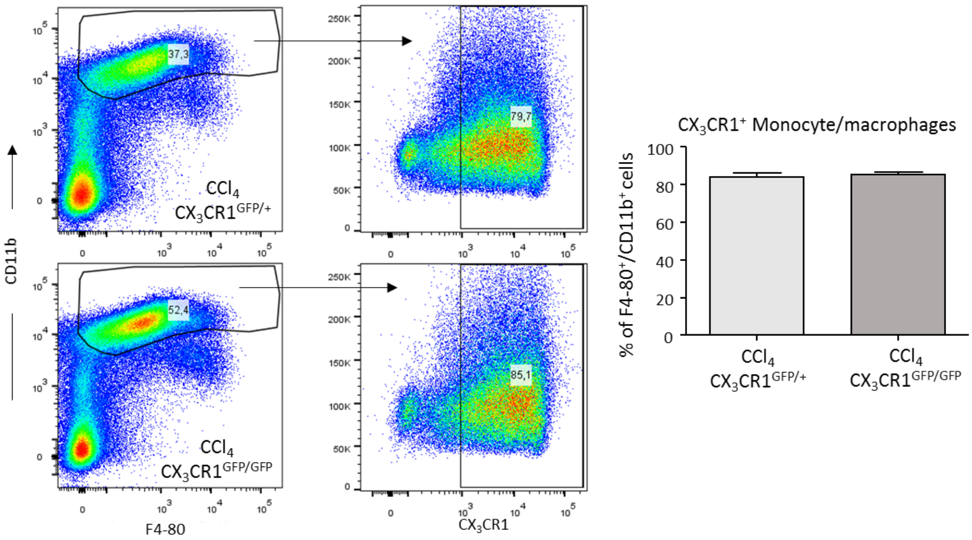

Supplementary Figure 3: The lack of CX_3CR1 did not interfere with the liver recruitment of CX_3CR1^+ monocytes/macrophages.

The distribution of F4-80$^+$/CD11b^{high}/CX_3CR1^+ monocytes/macrophages was evaluated by flow cytometry in the livers of $CX_3CR1^{gfp/+}$ and $CX_3CR1^{gfp/gfp}$ mice 36 hours after receiving an acute dose of CCl_4.

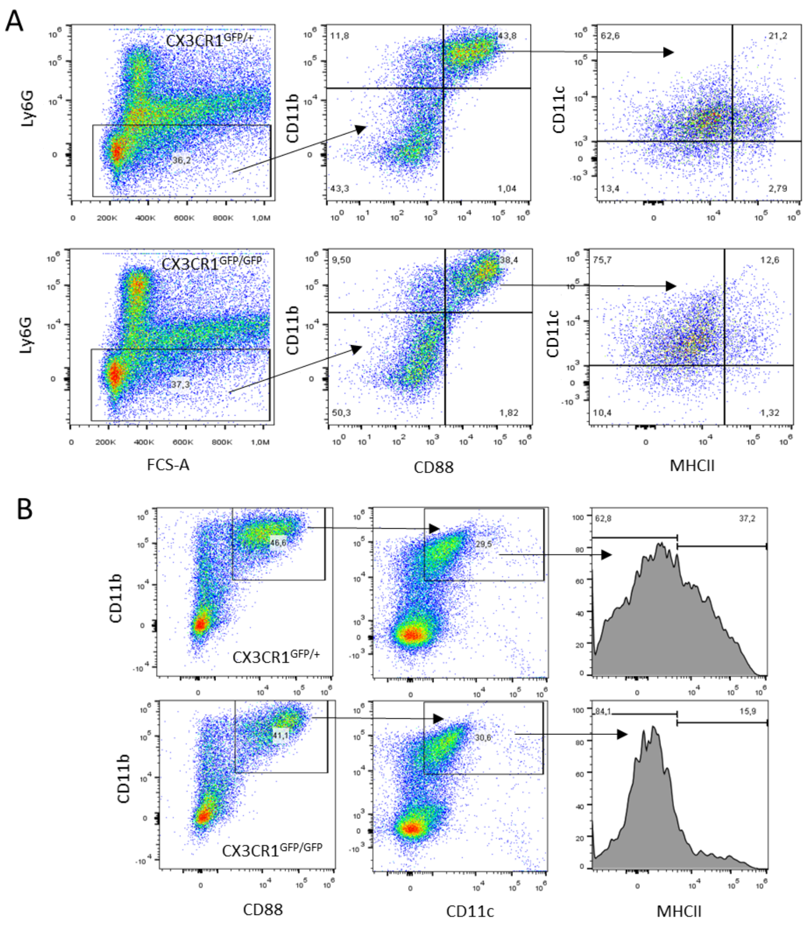

Supplementary Figure 4: The lack of CX_3CR1 affects *in vitro* differentiation of monocyte-derived dendritic cells (moDCs).

MoDCs were allowed to differentiate by 7 days culture of bone marrow myeloid cells from either $CX_3CR1^{gfp/+}$ and $CX_3CR1^{gfp/gfp}$ mice in RPMI-1640 medium supplemented with 10% fetal bovine serum. (Panel A) Effect of CX_3CR1 deficiency on the maturation of $Ly6G^-$/$CD11b^+$/$CD88^+$/$CD11c^+$/$MHCII^{high}$ moDCs. (Panel B) Effect of CX_3CR1 deficiency on the expression of MHCII by $CD11b^+$/$CD88^+$/$CD11c^+$ pre-dendritic cells.

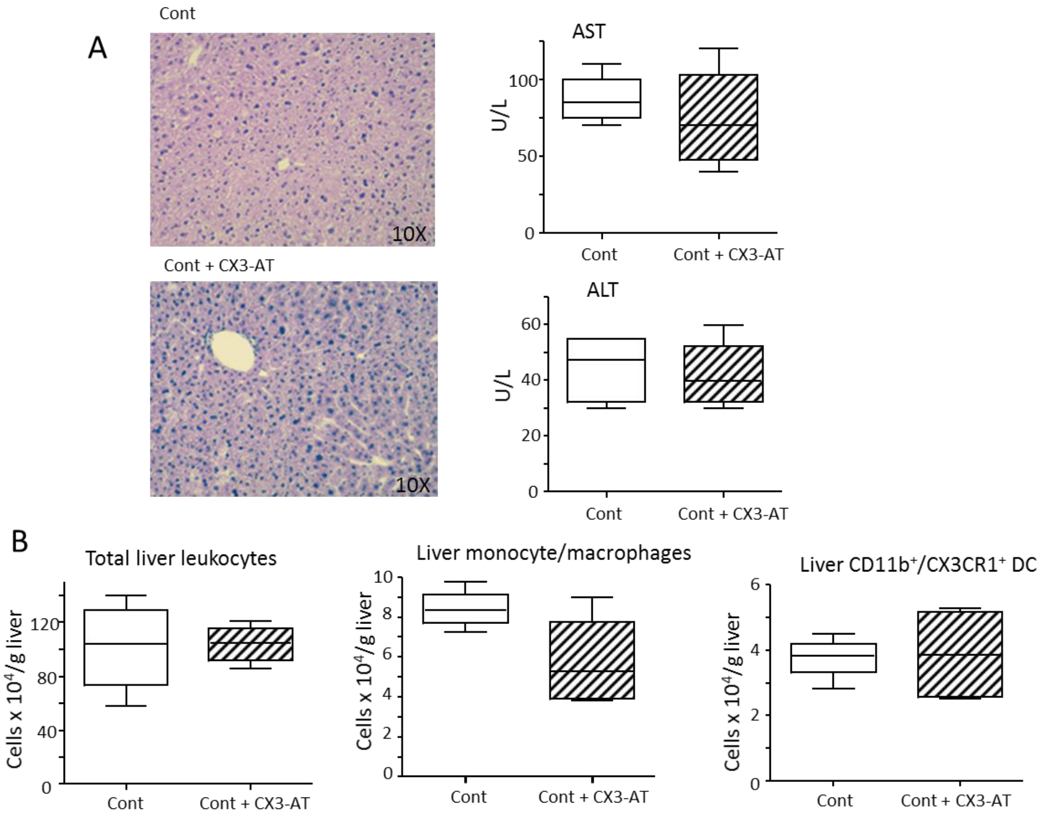

Supplementary Figure 5: Effect of CX_3CR1 antagonist CX3-AT in naïve mice.

Wild-type mice were injected IP with a single dose of CX3-AT (150 µg in saline) or saline (Cont) and the effects on liver integrity and hepatic leukocyte distribution were evaluated after 12 hours. (Panel A) Liver morphology (Hematoxylin/eosin staining of formalin-fixed liver sections) and circulating levels of alanine aminotransferase (ALT) and aspartate aminotransferase (AST). (Panel B) Flow cytometry count of total liver leucocytes ($CD45^+$ cells), monocyte/macrophages (F4-80^+/$CD11b^{high}$ cells) and CX_3CR1-expressing myeloid dendritic cells ($CD11b^+$/$CD11c^+$/$MHCII^{high}$ cells)- The values are expressed as means ±SD of three different cell preparations.

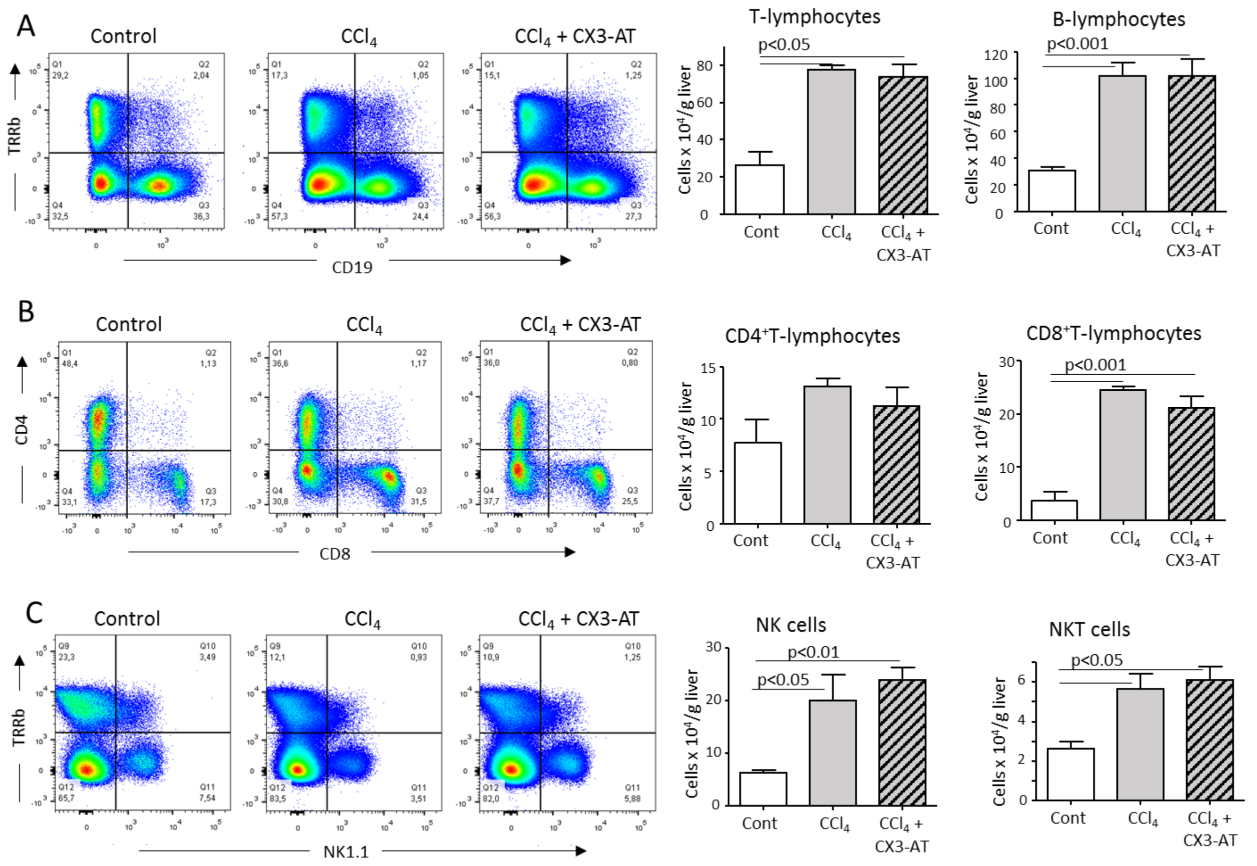

Supplementary Figure 6: CX3-AT does not affect the liver distribution of different lymphocyte sub-sets during hepatic inflammation.

Liver lymphocytes were analyzed by flow cytometry in naïve mice, in animals receiving CCl_4 alone and in combination with CX3-AT. (Panel A) Liver distribution of TCR-β^+/CD19$^-$ T- and TCR-β^-/CD19$^+$ B-lymphocytes. (Panel B) Liver distribution of CD4$^+$/CD8$^-$ helper and CD4$^-$/CD8$^+$ cytotoxic T-lymphocytes. (Panel C) Liver distribution of TCR-β^-/NK1.1$^+$ NK and TCR-β^+/NK1.1$^+$ NKT cells. The values are expressed as means ±SD of 5 different cell preparations.
